# Effects of adipose derived stem cells pretreated with resveratrol on sciatic nerve regeneration in rats

**DOI:** 10.1038/s41598-023-32906-9

**Published:** 2023-04-10

**Authors:** Ziqiang Zhang, Mengyu Zhang, Yingying Sun, Monan Li, Chenhao Chang, Weiqi Liu, Xuemin Zhu, Lan Wei, Fengyun Wen, Yumei Liu

**Affiliations:** 1 College of Animal Science and Technology, Henan University of Science and Technology, Luoyang, 471000 Henan China; 2 The School of Materials Science and Engineering, Henan University of Science and Technology, Luoyang, 471000 Henan China

**Keywords:** Cell biology, Stem cells

## Abstract

Adipose derived stem cells (ADSCs) are popular in regenerative medicine due to their easy availability, low immunogenicity and lack of controversy regarding their ethical debate use. Although ADSCs can repair nerve damage, the oxidative microenvironment of damaged tissue can induce apoptosis of transplanted stem cells, which weakens the therapeutic efficacy of ADSCs. Resveratrol (Res) is a type of natural polyphenol compound that regulates the proliferation, senescence and differentiation of stem cells. Therefore, we investigated whether incubation of ADSCs with Res improves their to promote peripheral nerve regeneration. ADSCs were cultured in vitro and treated with H_2_O_2_ to establish an apoptosis model. The control, H_2_O_2_ and Res groups were set up. The cell survival rate was detected by the CCK-8 method. The TUNEL assay was used to detect the apoptosis of the cells. qRT‒PCR was used to analyze the expression of apoptosis-related mRNA, and the effect of Res on the proliferation of ADSCs was investigated. In vivo, 40 SD rats were randomly divided into the control, model, ADSCs and ADSC + Res groups, with 13 rats in each group. The sciatic nerve injury rat model was established by the clamp method. Gait was observed on Days 7, 14, 21, and 28. Sciatic nerve regeneration was detected on Day 28. Res had no effect on the proliferation of ADSCs, and the TUNEL assay confirmed that Res pretreatment could significantly improve H_2_O_2_-induced apoptosis in ADSCs. Compared with the control group, caspase-3, Bax and Bcl-2 expression levels were significantly increased in the H_2_O_2_ group. Compared with the H_2_O_2_ group caspase-3 and Bax expression levels were significantly decreased, and Bcl-2 expression levels were significantly increased in ADSCs + Res group. At 4 weeks after surgery, the functional index of the sciatic nerve in the ADSCs + Res group was significantly higher than that in the model group. On Day 28, the average density of the sciatic nerve myelin sheath in the ADSCs + Res group was significantly increased compared with that in the model group, and Nissl staining showed that the number of motor neurons in the spinal cord was significant compared with that in the model group. Compared with the control group, the wet weight ratio of gastrocnemius muscle and muscle fiber area in ADSCs + Res group were significantly increased. Res enhanced the ability of ADSCs to promote sciatic nerve regeneration in rats.

## Introduction

Peripheral nerve injury is one of the most common traumatic burdens encountered in clinical practice^1^. For nerve defects and short gaps, end-to-end anastomosis is usually used to repair nerve damage. However, long-term peripheral nerve damage caused by traumatic injury usually requires transplantation of other nerves to fill the gap in the injury^2^. Although autologous nerve transplantation has obvious therapeutic effects, its application is impeded by high costs and limited donor nerve sources^3,4^. Seeking new therapeutic approaches to improve and especially accelerate axonal nerve regeneration is of great importance.

Stem cell therapy has emerged as a new potential therapy for repairing nerve damage. Mesenchymal stem cells (MSCs) are multifunctional cells derived from adipose tissue, bone marrow and umbilical cord. ADSCs are precursors obtained and processed from the body’s adipose tissue and are popular in regenerative medicine due to their accessibility, non-nonimmunogenicity and pluripotency^5^. Accumulating evidence has shown that ADSCs can differentiate into various cell types, including neurons and Schwann cell-like cells and there are studies indicating their beneficial effect on peripheral nerve regeneration after injury^6^ However, due to the low mobility and survival rate of transplanted cells in the pathological microenvironment of the nerve injury site, the therapeutic effect of stem cell-based therapy in promoting nerve regeneration is limited. Thus, it is imperative to enhance cell survival for nerve regeneration in nerve injury.

Pretreatment has been shown to be a rational approach for reinforcing cells to withstand the ischemia and reperfusion injury environment. Research has shown that pharmacological preconditioning of stem cells is a reasonable way to strengthen their defenses against damage in the injury environment^7,8^. Res is a naturally occurring small polyphenolic compound found in a variety of common foods, including grapes, blueberries and peanuts. Res has been reported to effectively influence oxidative stress, inflammation, mitochondrial dysfunction, apoptosis and angiogenesis^9^. Song^10^ et al. found that Res at a concentration of 0.01–10 μmol/L could promote the proliferation of mouse bone marrow mesenchymal stem cells (BMSCs) in a dose-dependent manner, accompanied by an increase in osteogenic potential. Cheng^11^ et al. also showed that Res could promote the survival and proliferation of neural stem cells in a concentration-dependent manner by activating Shh (Sonic Hedgehog) signaling. In addition, Res can promote in vitro culture and amplification of human umbilical cord blood derived hematopoietic stem cells by maintaining stem cell phenotypes^12^.

This study investigated whether preconditioning ADSCs with Res could protect them from H_2_O_2_-induced injury. In addition, to confirm their therapeutic applicability, we explored the effect of Res-pretreated ADSCs transplantation on sciatic nerve regeneration in rats.

## Materials and methods

### Isolation and culture of ADSCs and preincubation with Res

Briefly, the inguinal fat pad was removed from the abdominal cavity of rats anesthetized with diethyl ether. The adipose tissue was digested at 37 °C for 70 min using type I collagenase (Sigma, USA). After centrifugation at 170 × g for 10 min, the cells were resuspended in the lower layer using DME/F12 (HyClone, USA), and then filtered through a 70 μm mesh. The collected cells were cultured in a 37 °C, 5% CO_2_ incubator in DME/F12 containing 10% FBS (Gibco, USA). After 48 h, the medium was changed for the first time. When the cells reached confluence, the adherent cells were detached with trypsin/ethylenediaminetetraacetic acid (EDTA) (Sigma, USA) and reseeded for expansion^13^. When the ADSCs reached the third generation, Res at a concentration of 20 μM was added and cells were preincubated for 24 h for subsequent experiments.

### Multidirectional differentiation of ADSCs

Rat ADSCs were inoculated in 6-well plates with a cell density of 1 × 108 cells /L. When the cell growth reached 80–90%, the complete medium was changed into lipid or osteogenic induction medium for lipid culture for 12–14 days^14^, and bone culture for 19–21 days^15^, and oil red O dye and alder red staining were performed, respectively.

### H_2_O_2_-induced oxidative stress model

Harvested ADSCs were incubated in DMEM/F12 medium containing serum for 12 h before being treated with 300 μM H_2_O_2_ for 4 h. Then, a variety of methods were used to detect the cellular apoptosis in each group to determine the appropriate concentration of H_2_O_2_ needed in our model.

### Cell proliferation assay

ADSC proliferation was assessed using the Cell Counting Kit 8 (CCK-8) assay (Beyotime Biotechnology, China). According to the manufacturer's protocol, the cells were seeded in 96-well plates at 5 × 10^4^ cells/mL cell density, and 100 μl per well was added. After the cells adhered to the wall, 10 μl of CCK-8 solution was added to each well. After incubation for 4 h, the absorbance at a wavelength of 450 nm per well was measured using a microplate reader (Multiskan FC, Thermo). The cell survival rate was calculated using the following formula: survival rate (%) = (OD_Treatment group_ − OD_Blank group_)/(OD_Control group_ − OD_Blank group_).

### RNA extraction and qRT‒PCR

Total RNA was extracted from ADSCs using a miRNA isolation kit (Tiangen, Beijing, China) according to the manufacturer's instructions. qRT‒PCR measured Bax, Bcl-2 and Caspase-3 expression in CFX Connect TM Real-Time System (BioRad). β-actin was used as the normal control. Data were analyzed by the 2^−△△Ct^ method. Primer sequences (Sangon Biotech, Shanghai, China) are shown in Table 1.

**Table 1:** Primer sequences.

Gene	Primer sequence (5′–3′)
Bax	Forward: AGA AGC TGA GCG AGT GTC TC
	Reverse: GTG TCC AGC CCA TGA TGG TT
Bcl-2	Forward: GAG TGG GAT ACT GGA GAT GAA GAC
	Reverse: GAG AAG TCA TCC CCA GCC C
Caspase-3	Forward: ACT GGA ATG TCA GCT CGC A
	Reverse: TTT TCA GGT CCA CAG GTC CG
β-actin	Forward: TGA CAG GAT GCA GAA GGA GA
	Reverse: TAG AGC CAC CAA TCC ACA CA

### Terminal deoxynucleotidyl transferase dUTP nick end labeling (TUNEL) staining

Cells were immobilized in 4% paraformaldehyde for 60 min at 37 °C and 0.5% Triton X-100 for 10 min. The cells were washed with PBS 3 times and incubated with a cell death detection kit (Roche, Basel, Switzerland) according to the manufacturer’s instructions. Cell nuclei were counterstained with 0.1 g/mL 4 ,6-diamidino-2-phenylindole (Beyotime, Nantong, China), and imaging was conducted under microscope.

### Establishment of the sciatic nerve injury model

**Figure 1:**
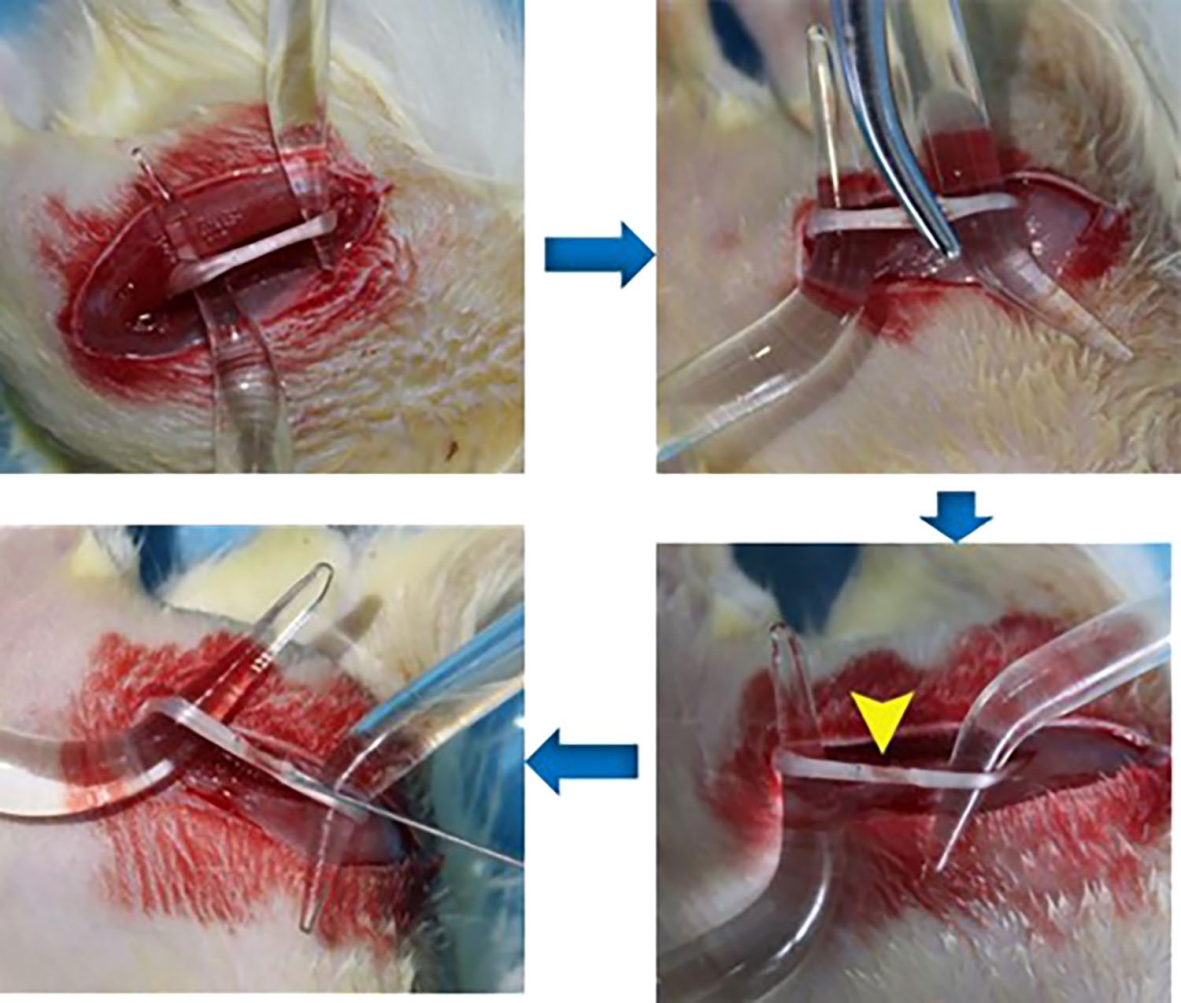
Establishment and treatment of sciatic nerve injury model in SD rat (arrow indicates injury).

(1) After the rats were weighed, the dosage of chloral hydrate (0.5 ml/kg) was calculated. After chloral hydrate was extracted by a syringe, the rats were anesthetized by intraperitoneal injection. (2) After the hair was removed from the operation area of the left leg by a shaving machine, the rats were moved to a sterile environment for surgery. The rats were placed on their side, fixed, disinfected with iodophor, and deiodized with alcohol. (3) The rat gluteal muscle was cut open with a scalpel, and the sciatic nerve was exposed. (4) After the sciatic nerve was picked out with a glass minute hand, the nerve was tightly squeezed for 10 s with 5-bend hemostatic forceps, and this was repeated 3 times with an interval of 10 s. The nerve color became translucent and the injury was deemed successful. (5) At the end of the test, the nerves were returned to their original place after marking the initial proximal end of the injury with a 10–0 ophthalmic shovel needle. (6) Rinse the wound with 0.9% normal saline, suture layer by layer, and gently apply erythromycin ointment to each layer to prevent infection (Fig. 1).

### Animals and sciatic nerve therapy

Sprague‒Dawley (SD) rats (180‐220 g) were provided by the Experimental Animal Center of Henan University of Science and Technology. Study findings have been reported in accordance with ARRIVE guidelines. Rats were randomly divided into four groups: (1) Control group: sham + PBS; (2) Model group: crush + PBS; (3) ADSCs group: crush + ADSCs; and (4) Res + ADSCs group: crush + Res preincubated ADSCs. Next, sterile PBS solution, a cell suspension of 1 × 10^6^ cells ADSCs, or a cell suspension of 1 × 10^6^ cells Res-incubated ADSCs (24 h incubation) were injected into rats with sciatic nerve clipping injury using microsyringes in a 10 μL volume. Then, the wound was closed by suture. All animal protocols were approved by the Guide for the Care and Use of Laboratory Animals published by the US National Institutes of Health. In addition, all experimental procedures strictly abided by the requirements of the ethics committee of Henan University of Science and Technology. Third generation ADSCs were used for treatment.

### Sciatic functional index (SFI)

The sciatic nerve function index (SFI)^16^ was analyzed at 1, 2, 3, and 4 weeks after treatment. A piece of white paper (15 × 50 cm) was placed into a cardboard box with open ends (length = 70 cm, width = 15 cm, height = 15 cm) and a rat with its feet covered in ink was placed at the entrance of the cardboard box. The rat will walked right through the cardboard box, leaving footprints.

$$  {\text{SFI}} =  - 38.3\left( {{\text{EPL - NPL}}} \right)/{\text{NPL}} + 109.5\left( {{\text{ETS - NTS}}} \right)/{\text{NTS}} + 13.3\left( {{\text{EIT - NIT}}} \right)/{\text{NITT}} - 8.8  $$

The normal side (N), surgical side (E), first toe to fifth toe (TS), the second toe to fourth toe (IT), and third toe to heel (PL) were used to calculate SFI. An SFI value of -100 indicates total damage, while a value of 0 is considered normal for total damage.

### Luxol fast blue

The rats were euthanized on the 28th day after operation. Tissue samples were randomly cut and soaked in 4% paraformaldehyde for 48 h and then embedded in paraffin. Nerve sections were deparaffinized in xylene (2 × 5 min), hydrated in graded ethanol (2 × 5 min in 100%, 5 min in 85%, and 5 min 70%) followed by distilled water and finally rinsed in PBS. Next, the sections were stained overnight at 56 °C in 0.1% Luxol fast blue (LFB) (Sigma, St. Louis, MO, USA) in acidified 95% ethanol, rinsed in 95% ethanol and differentiated in 95% ethanol. For the detection of the percentage of myelin area in rats, we randomly selected three rats from each group, and each rat made two sections for Luxol Fast Bule staining and scanning. The scanned images were enlarged to 50 μm by Caseviewer software and then five faces were randomly selected. The percentage of myelin area of rats was calculated, Image-Pro Plus software was used to calculate and the histogram was made by PRISM software.

### Gastrocnemius muscle weight analysis and H&E staining

On the 28th day after surgery, the normal hind limbs and the gastrocnemius muscle of the operative hind limbs of rats in each group were weighed.

$$ {\text{Gastrocnemius wet weight ratio }}\left( \% \right) \, = {\text{ Operative side}}/{\text{Normal side }}*{1}00\% . $$

Three rats in each group were randomly selected for sampling, and two tissue sections in each group were used for staining. The gastrocnemius abdominal specimen was fixed in 4% paraformaldehyde for 24 h, embedded in paraffin, cut into 5 mm slices and stained with H&E. After scanning, the Caseviewer software randomly captures five areas at a certain magnification rate of 100 μm. Five random areas were used for imaging of two samples in each group, and percentage collagen fiber analysis was performed using Image-Pro Plus. Finally, the histogram is generated using Prism software.

### Nissl staining

Spinal cord samples were collected at 28 d following surgery. Three rats in each group were randomly selected for detection of motor neurons in spinal cord cross-sectional areas. The spinal cord tissues collected from each rat were sliced to into 5‐µm sections, immersed in xylene, 100% ethanol, 75% ethanol, and distilled water for 3 min each, and then stained with a 1% toluidine blue solution at 56 °C for 20 min. The sections were washed in distilled water and differentiated in 1% glacial acetic acid for 2 min, immersed in 95% ethanol, soaked in anhydrous ethanol, and anhydrous ethanol, xylene, sealed with neutral gum and observed under a microscope. Two sections were cut into each rat for Nissl staining and scanning. The scanned image was enlarged to 200 μm with Caseviewer software, and five randomly selected areas were enlarged to 100 μm before taking a screenshot. The number of motor neurons was calculated and counted by Image-Pro Plus software, and the bar chart was made by PRISM software.

### S-100/NF-200 double standard immunofluorescence staining

Four weeks after the operation, the fixed nerve was put into the solution prepared in advance and placed at 4 ℃ to ensure the tissue sink to the bottom of the bottle. The tissue was sectioned with a frozen slicer with a thickness of 16 μm.

The steps of the S-100/NF-200 immunofluorescence double-standard staining are as follows:

1. The sample was treated with 1% Triton X-100 in an incubator for 12 min.
2. It was sealed with 3% BSA blocking solution at room temperature to prevent nonspecific staining.
3. The sample was incubated with rabbit-resistant rat S-100 primary antibody (1∶100) and murine anti-rat NF200 primary antibody (1∶100) at 4 ℃ for 24 h.
4. A mixed solution of goat anti-mouse FITC secondary antibody (1∶200) and goat anti-rabbit TRITC secondary antibody (1∶200) was added slowly at room temperature for 2 h.
5. A sealing film was applied.
  In (1)–(4), after all operations, sterile PBS should be used before the next step can be carried out.
6. Images were taken and observed. Each group was randomly divided into three mice, each mouse and two slices were taken for observation. The scanned images were enlarged to 50 μm by Caseviewer software, and 5 regions were randomly selected for statistical analysis.
7. Image-Pro Plus software was used for calculations and the histogram was generated by using PRISM software.

### Statistical analysis

Data are expressed as the means ± standard deviations. Statistical analysis was performed using one-way ANOVA with SPSS 17.0 statistical software. A P value of less than 0.05 was considered statistically significant.

### Ethics approval and participation consent

All the procedures performed in the studies including animal care and euthanization, were approved by Henan University of Science and Technology (China) (No. 20200605023).

### ARRIVE guidelines

The study is reported in accordance with the ARRIVE guidelines.

## Results

### Characteristics of ADSCs

**Figure 2:**
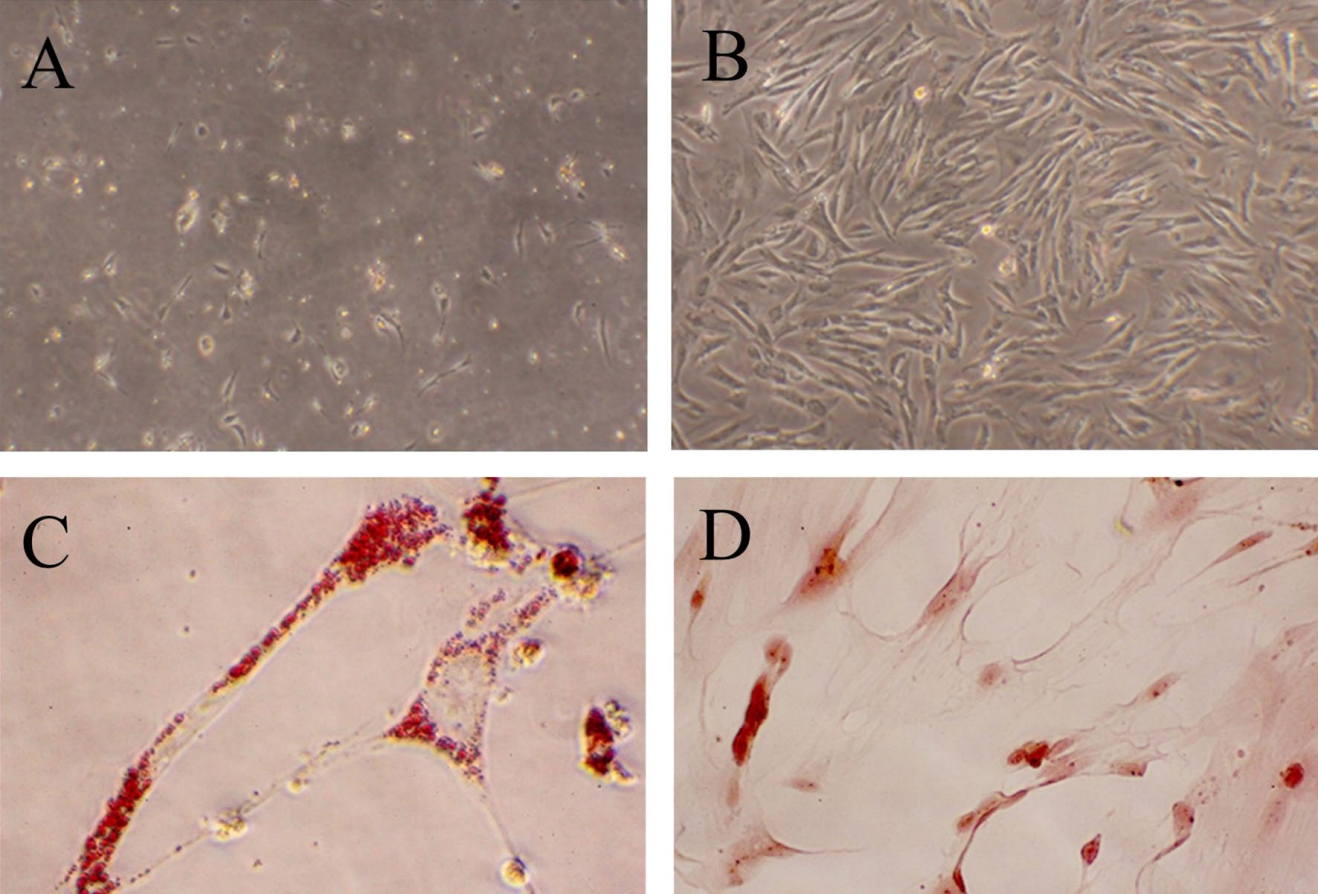
Isolation and identification of ADSCs. (**A**) Primary ADSCs (× 100); (**B**) Three generations ADSCs (× 100); (**C**) Oil Red O staining (× 400); (**D**) Alizarin Red Staining (×400).

After 24 h of rat ADSCs primary cell culture, the cells showed short spindle shape, aggregation and growth under the microscope, and the degree of adhesion was not tight (Fig. 2A). The volume of the third-generation ADSCs increased, and local Adscs grew in fusiform, clusters, and spirals, with rapid proliferation (Fig. 2B). After induction of lipid differentiation, lipid droplets were stained red by oil red O staining (Fig. 2C). After osteogenic differentiation was induced, alizarin red staining stained the internal calcium deposits of rat ADSCs to orange red (Fig. 2D).

### Effects of H_2_O_2_ on ADSCs

**Figure 3:**
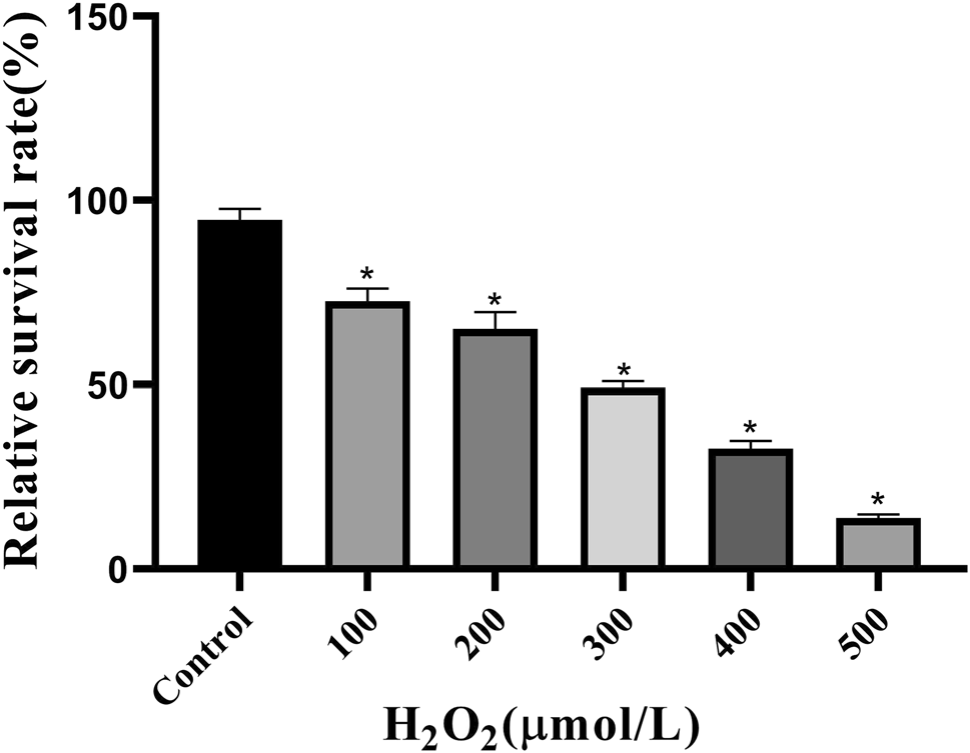
Effects of H_2_O_2_ (0, 100, 200,300, 400 and 500 μM) on ADSCs. *P < 0.05 compared with the control group, *P* < 0.05, n = 5.

To establish an apoptotic model of rat ADSCs, H_2_O_2_ (100, 200, 300, 400 and 500 μM) was selected to stimulate cells for 4 h. The viability of the cells was assessed using a microplate reader after incubation with the CCK-8 reagent. The survival rates of ADSCs in the 100, 200, 300, 400 and 500 μM H_2_O_2_ groups were 72.6 ± 3.45%, 65.1 ± 4.49%, 49.2 ± 1.76%, 32.7 ± 2.06%and 13.8 ± 1.03%, respectively, compared with the control group (*P* < 0.05) (Fig. 3). Compared with other groups, the 4 h treatment with 300 μM H_2_O_2_ caused approximately 50% apoptosis in ADSCs and was thus more suitable for the establishment of an in vitro apoptosis model.

### Res prevents H_2_O_2_-induced growth inhibition in ADSCs.

**Figure 4:**
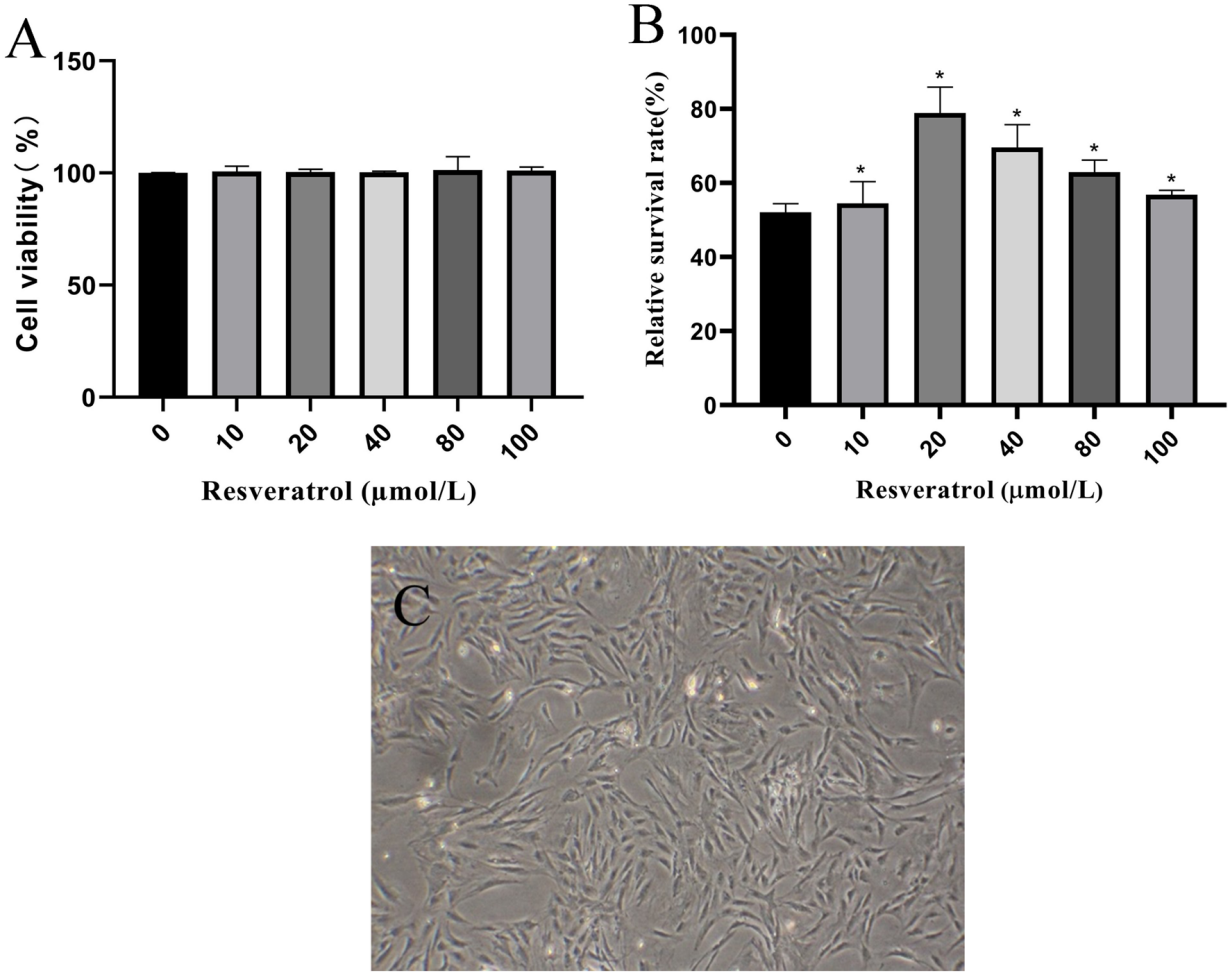
Drug concentration of ADSCs pretreated with Res (× 100). (**A**) Dynamic changes after 24 h of ADSCs treatment with different concentrations of Res (0, 10, 20, 40, 80 and 100 μM). (**B**) Effects of Res (0, 10, 20, 40, 80 and 100 μM) on cell viability in ADSCs induced by H_2_O_2_. (**C**) Resveratrol pretreated ADSCs for 24 h. *P < 0.05 compared with the control group, n = 5; #* P* < 0.05 compared with the 10 μM group.

The morphology of Res pretreated ADSCs under microscope was the same as that of untreated ADSCs (Fig. 4C). As shown in Fig. 4A, resveratrol alone (10–100 μM) for 24 h had no remarkable effect on the survival of ADSCs. In the next step, to investigate the protective effect of Res, ADSCs pretreated with Res for 24 h at concentrations ranging from 10 to 100 μM were exposed to 300 μM H_2_O_2_ for 4 h and the viability was analyzed by CCK-8 assay (Fig. 4B). H_2_O_2_-induced apoptosis was blocked in 10 to 100 μm Res concentrations compared to 0 μM Res. The survival rates of ADSCs in the 10, 20, 40, 80 and 100 μM res were 54.5 ± 5.91%, 78.9 ± 6.99%, 69.6 ± 6.17%, 62.9 ± 3.28% and 56.8 ± 1.17%, respectively. SPSS software was used to calculate the differences between different concentration groups, and it was found that 20 μmol/L Res was significantly different from 10 μmol/L Res, but not from other groups. However, the cell viability of ADSCs incubated with 20 μmol/LRes increased by 30.93%, 11.79%, 20.28% and 28.01% compared with 10 μmol/L, 40 μmol/L, 80 μmol/L and 100 μmol/L, respectively. The cell survival rate of 20 μmol/L Res and 0 μmol/L Res groups was significantly different. Therefore, ADSCs pretreated with 20 μM Res could better resist H_2_O_2_-induced apoptosis.

**Figure 5:**
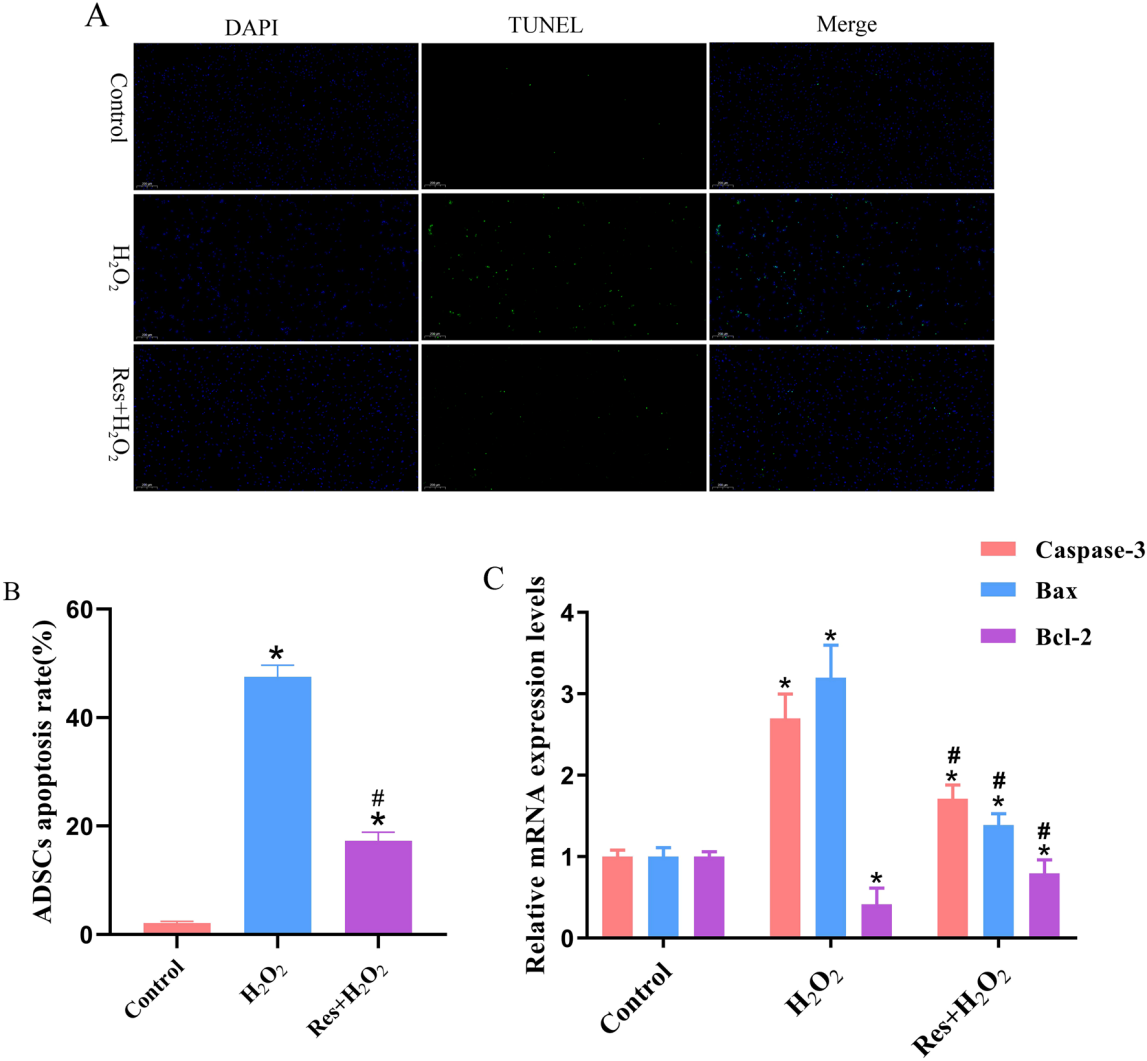
Effects of Res pretreatment on the activity of H_2_O_2_-induced ADSCs. (**A**) TUNEL staining was used to detect the apoptosis rate of ADSCs induced by H_2_O_2_ after 24 h Res treatment. (**B**) The apoptosis rate of the ADSCs in (**A**). (**C**) Effects of Res on the expression of mRNA in H_2_O_2_-induced ADSCs. *P < 0.05 compared with the control group, n = 5. #*P* < 0.05 compared with the H_2_O_2_ group, n = 5. Scale bar: 50 μm.

Compared with the control group, expression levels of the apoptosis-related proteins Caspase-3 and Bax in H_2_O_2_ group were significantly upregulated by 1.69 fold and 2.19 fold, respectively, while the expression levels of Bcl-2 were significantly decreased by 0.413 fold. Compared with the H_2_O_2_ group, Caspase-3 and Bax expression levels in the Res + H_2_O_2_ group were significantly downregulated by 0.98 fold and 0.81 fold, respectively, while the expression levels of Bcl-2 were significantly increased by 0.92 fold. (Fig. 5C, P < 0.05). the TUNEL assay further confirmed that Res + H_2_O_2_ group could effectively improve H_2_O_2_ -induced ADSC apoptosis (Fig. 5A,B). Compared with the control group, the apoptosis rate of the H_2_O_2_ group was significantly increased by 45.37%. Compared with the H_2_O_2_ group, the apoptosis rate of the Res + H_2_O_2_ group was significantly increased by 30.23%.

### ADSCs pretreated with Res can promote sciatic nerve function recovery.

**Figure 6:**
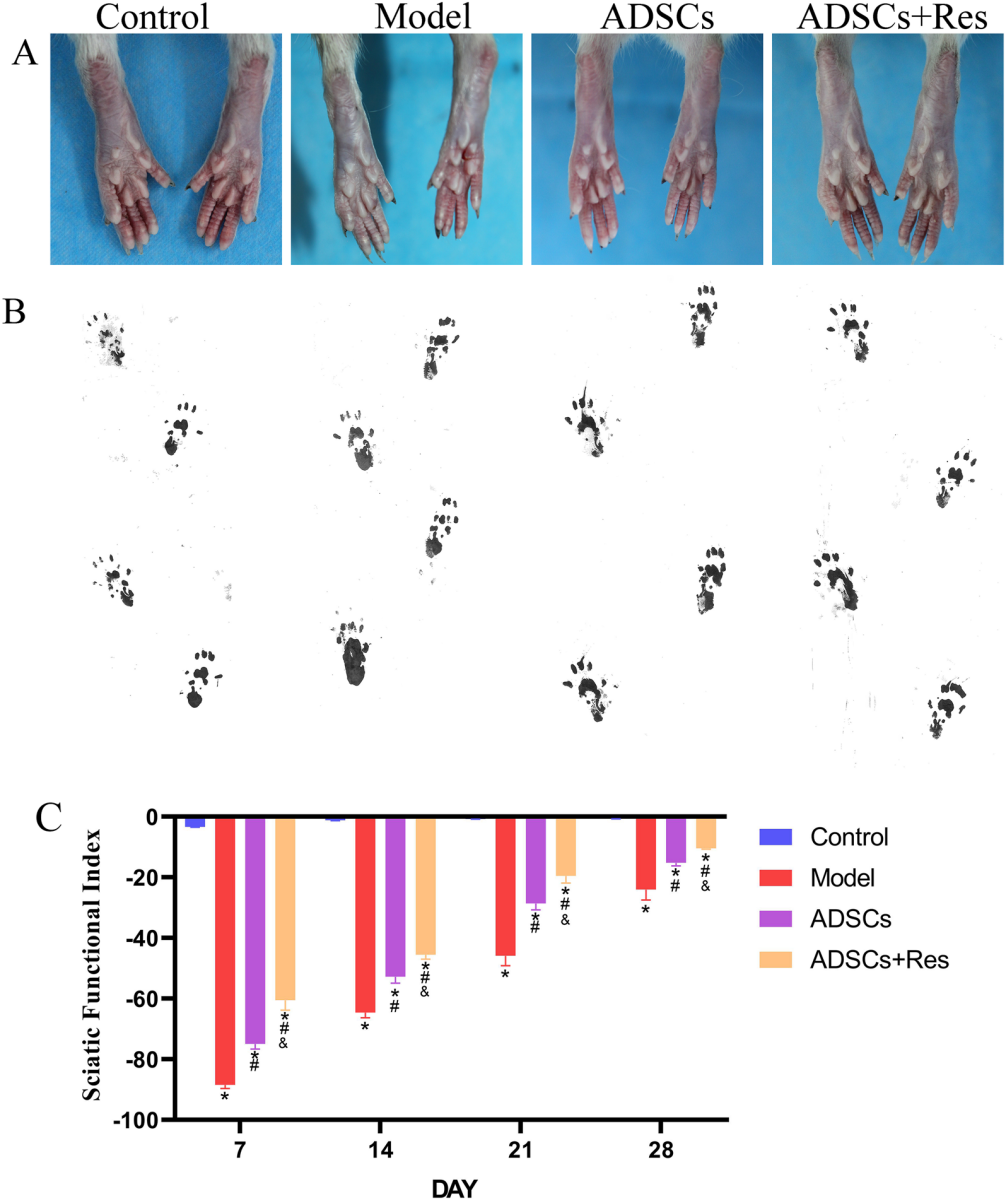
Effects of ADSCs pretreated with resveratrol on functional recovery after sciatic nerve injury. (**A**) Recovery of foot morphology; (**B**) Rat footprint; (**C**) SFI scores at different time points for the functional recovery from peripheral injury of the rats. *P < 0.05 compared with the control group, n = 5; #P < 0.05 compared with model group, n = 5.

SFI was calculated using Bain's formula to evaluate sciatic nerve regeneration in rats. Footprints and the results of each group are shown in Fig. 6. At 1 week after operation, the SFI of the model group was significantly reduced by 7.74 times compared with that of the control group. After treatment, the SFI of ADSCs group and ADSCs + Res group increased significantly by 15.23% and 31.58% compared with the model group, respectively. The SFI of ADSCs + Res group was 19.29% higher than that of ADSCs group. At 2 weeks after operation, the SFI of the model group was 63.36 lower than that of the control group. After treatment, the SFI of ADSCs group and ADSCs + Res group was significantly increased by 18.22% and 29.49% compared with the model group, respectively. SFI in ADSCs + Res group was significantly increased by 13.79% compared with ADSCs group. At 3 weeks after operation, the SFI in the model group was 45.28 lower than that in the control group. After treatment, the SFI of ADSCs group and ADSCs + Res group was significantly increased by 37.64 and 57.39% compared with the model group, respectively. SFI in ADSCs + Res group was significantly increased by 31.67% compared with ADSCs group. At 4 weeks after operation, the SFI of the model group was 23.45 lower than that of the control group. After treatment, the SFI of ADSCs group and ADSCs + Res group was significantly increased by 36.69% and 56.58% compared with the model group, respectively. SFI in ADSCs + Res group was significantly increased by 31.41% compared with ADSCs group. The results of four-week treatment showed that the SFI of the weekly ADSCs group and the ADSCs + Res group increased significantly, and the SFI of the ADSCs + Res group increased more significantly than that of the ADSCs treatment group. At 1 w after the operation, the SFI in the model group was significantly lower than that in the control group. At 1, 2, 3, and 4 weeks after the operation, the neurological function of rats in the weekly ADSCs group and ADSCs + Res group was significantly higher than that in the model group (*P* < 0.05), and the SFI value of the regenerative nerve in ADSCs + Res group was better than that in the ADSC group every week (*P* < 0.05), the treatment effect of ADSCs + Res group was the most significant at week 4, which was most similar to that of the control group (Fig. 6). Therefore, Res preconditioning of ADSCs accelerates the functional recovery after sciatic nerve injury in rats.

### Effects of ADSCs pretreated with Res on myelin regeneration after sciatic nerve injury in rats

**Figure 7:**
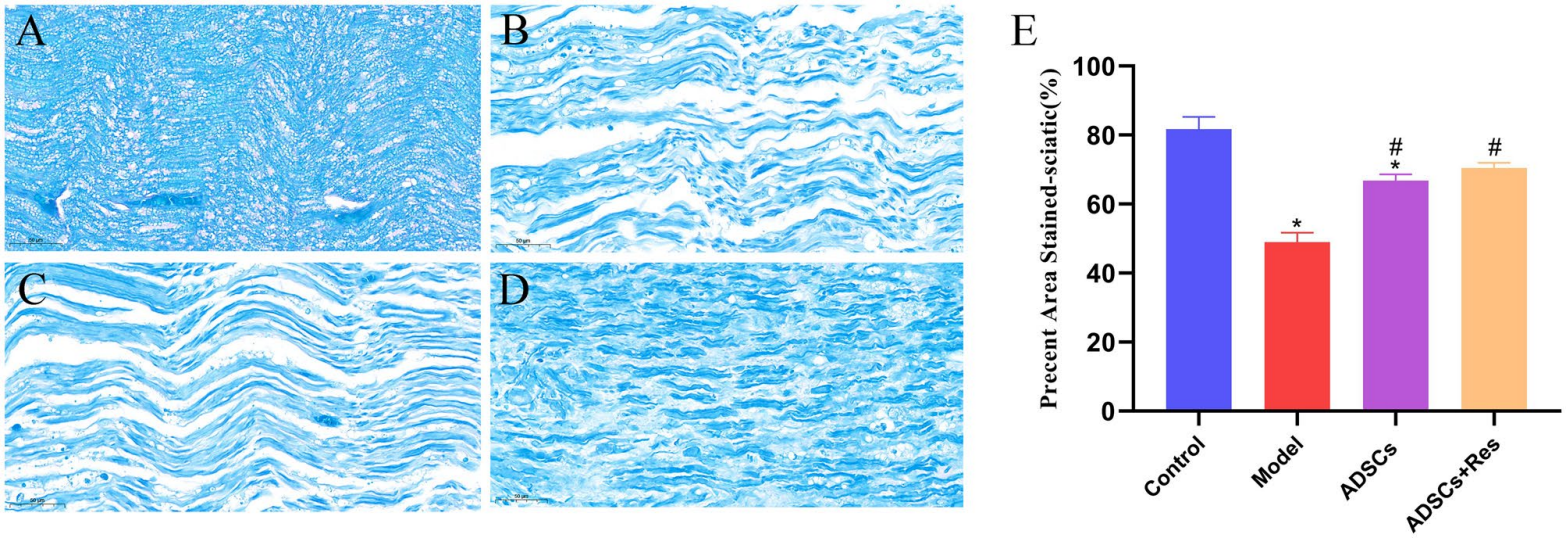
Effects of ADSCs pretreated with resveratrol on myelin regeneration after sciatic nerve injury in rats. (**A**) Control group; (**B**) Model group; (**C**) ADSCs group; (**D**) ADSCs + Res group; (**E**) Percentage of the stained sciatic area. *P < 0.05 compared with the control group, n = 5; #*P* < 0.05 compared with model group, n = 5. Scale bar: 50 μm.

To evaluate whether Res-pretreated ADSCs could promote nerve fiber regeneration and remyelination, morphological analysis of the injury distal site was performed 4 weeks after surgery. As shown in Fig. 7, the sciatic nerve structures of the control group were stained with Luxol fast blue (LFB) and showed uniform myelin staining without demyelination. In the model group, myelin was broken and atrophied, and axons were swollen and atrophied. Improvements were seen in all treatment groups. At the same time, compared with the control group, the percentage of staining area in the model group was significantly reduced by 32.708% (*P* < 0.05). Compared with the model group, the ADSCs group and ADSCs + Res group increased by 17.815% and 21.463%, respectively (*P* < 0.05). The percentage of sciatic nerve staining area in ADSCs + Res group was 3.648% higher than that in the ADSC group, and the treatment effect of the ADSCs + Res group was better.

### ADSCs pretreated with Res can alleviate muscle atrophy

**Figure 8:**
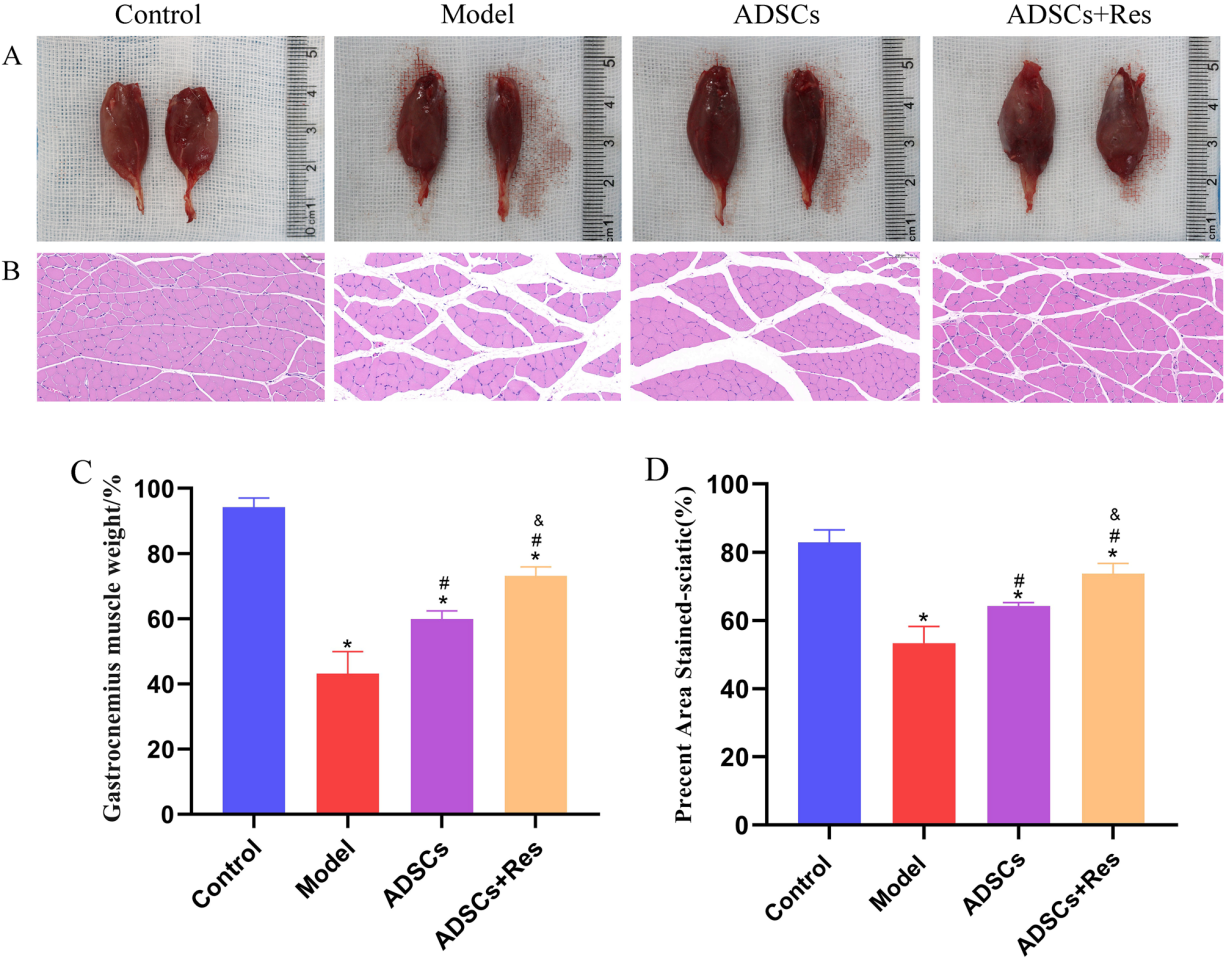
Histological assessment of the gastrocnemius muscle. (**A**) Image of the normal and surgical side of the gastrocnemius; (**B**) HE staining of the cross section of the gastrocnemius; Scale bar: 100 μm. (**C**) Statistical results of gastrocnemius wet weight ratio; (**D**) Muscle fiber cross-sectional area statistical results. *P < 0.05 compared with the control group, n = 5; #*P* < 0.05 compared with model group, n = 5; &*P* < 0.05 compared with ADSCs group, n = 5.

The muscle mass of gastrocnemius specimens was measured and HE staining was performed at 4 w. As shown in Fig. 8, the wet weight ratio of the gastrocnemius muscle in the control group was 94.328 ± 4.17% at 28 days after surgery, while that in the model group was significantly reduced to 43.215 ± 1.99%. The wet-weight ratio of the ADSCs and ADSCs + Res groups was 1, and 2 respectively, which was significantly higher than that of the model group (P < 0.05). H&E staining showed that compared with the control group, the muscle fiber area in the model group was significantly reduced by 29.591 (*P* < 0.05). Compared with model group, muscle fiber area in the ADSCs group and ADSC + Res group increased by 10.905% and 20.373%, respectively. Notably, the muscle fiber area of ADSCs + Res group was significantly increased by 9.468% compared with that of the ADSCs group (*P* < 0.05).

### ADSCs pretreated with Res decrease motor neuron loss in injured sciatic nerves

**Figure 9:**
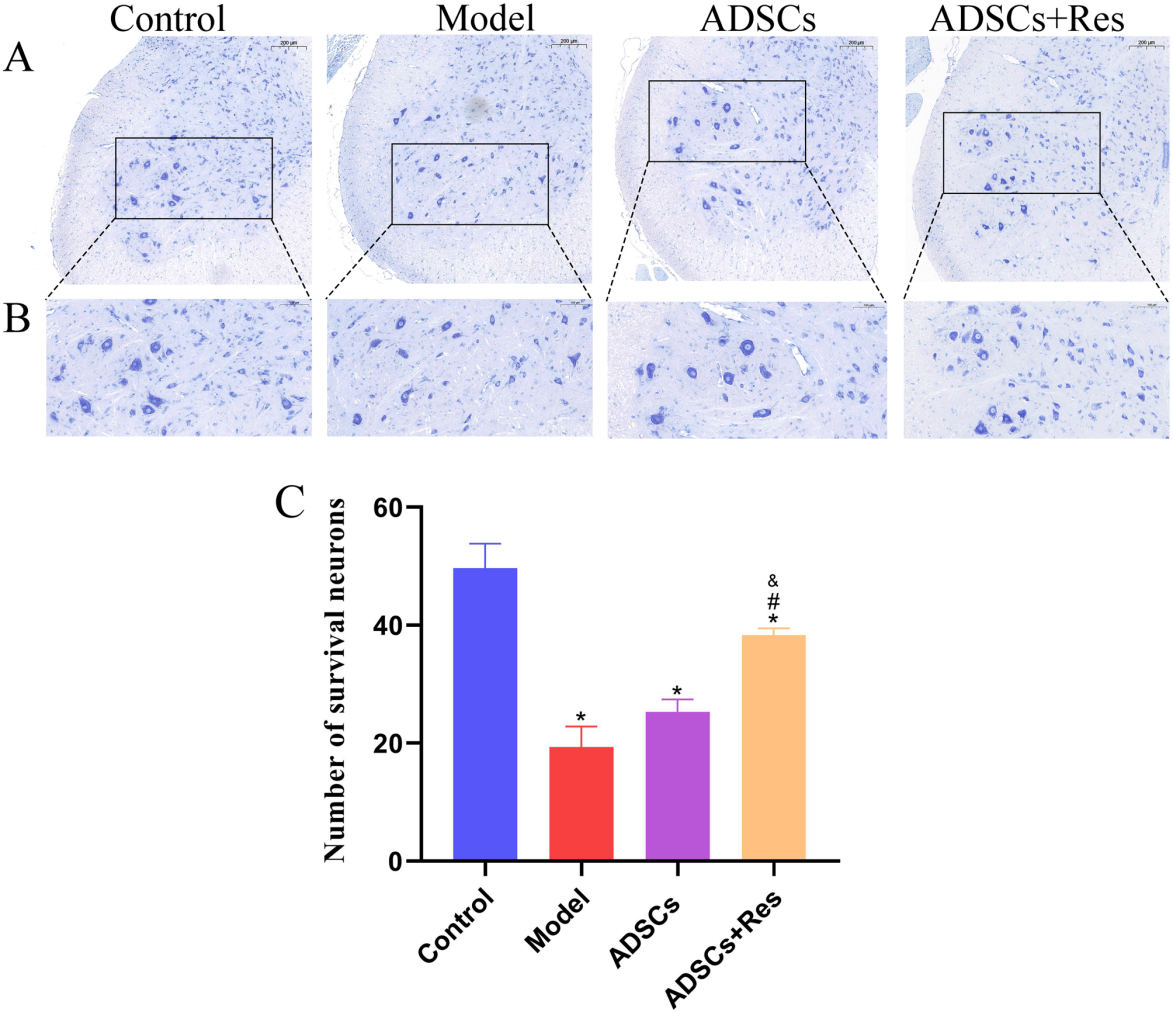
Histological assessment of the spinal cord. (**A**, **B**) L4 anterior horn Nissl staining; (**C**) The number of intact motor neurons in each group. *P < 0.05 compared with the control group, n = 5; #*P* < 0.05 compared with the model group, n = 5; and *P* < 0.05 compared with the ADSCs group, n = 5. Scale bar: 200 μm.

Sciatic nerve injury can cause ventral neuron necrosis and apoptosis. Nissl staining was performed on spinal cord transects 4 w after sciatic nerve injury to investigate the potential neuroprotective effects of Res preconditioning of ADSCs, as shown in Fig. 9. Nissl staining showed that compared with the control group, the volume of nerve cells in the model group was reduced, the cytoplasm was loose, and the nucleus was pyknotic. The number of intact motor neurons was significantly lower than 61.074% in the control group (*P* < 0.05). Compared with the model group, the number of intact motor neurons in the ADSC group and ADSCs + Res group was increased (*P* < 0.05).

### Resveratrol pretreatment of ADSCs can enhance the expression of S-100/NF-200 after sciatic nerve injury

**Figure 10:**
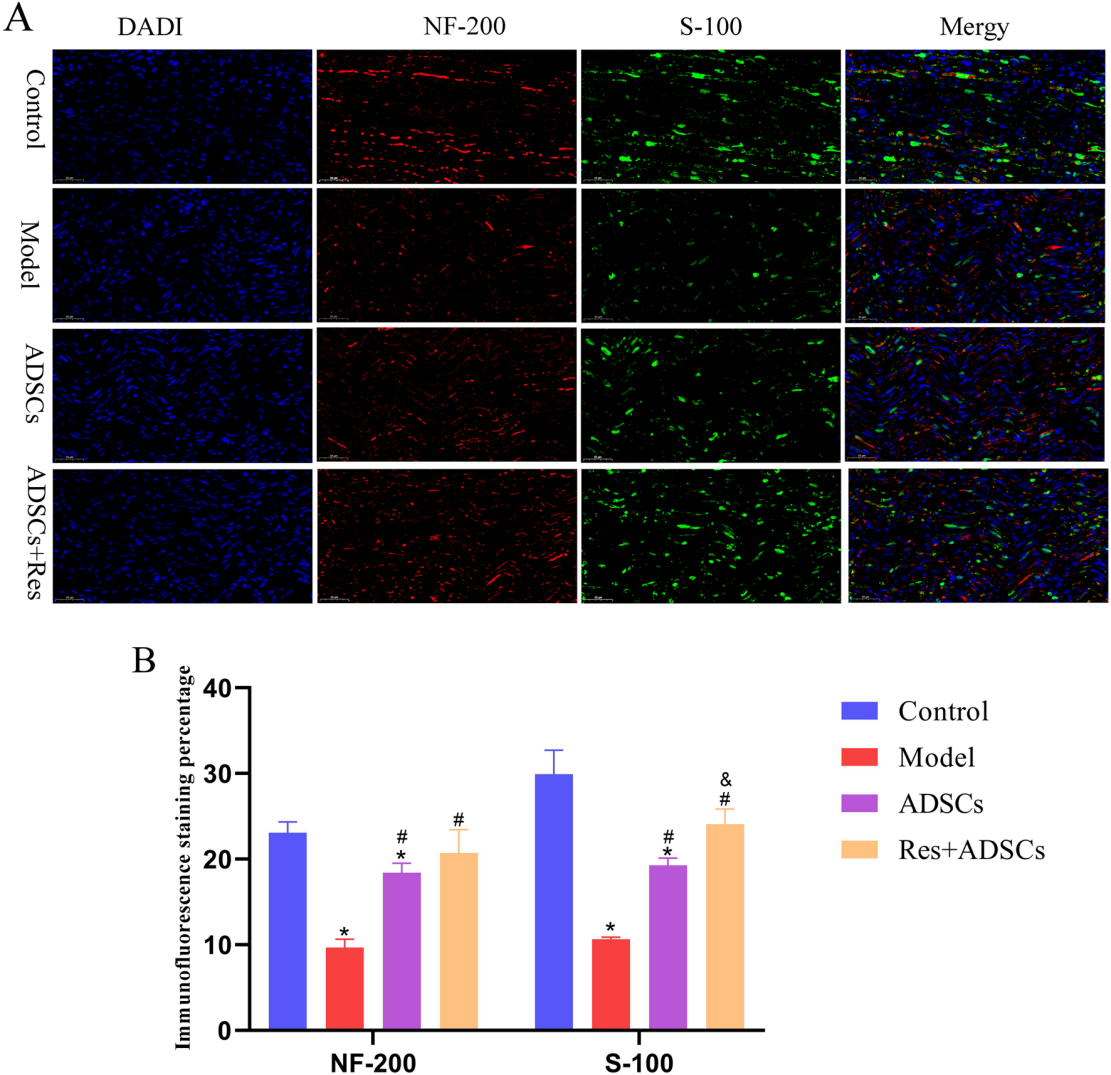
Effect of Res preconditioned ADSCs on S-100/NF-200 expression after sciatic nerve injury in rats. (**A**) Sciatic nerve S-100 /NF-200 immunofluorescence double standard staining; (**B**) Immunofluorescence staining percentage; * Indicates a significant of difference compared with control group, *P* < 0.05, n = 5. # Indicates a significant of difference compared with the model group, *P* < 0.05, n = 5 and Indicates a significant of difference compared with the ADSC group, *P* < 0.05, n = 5. Scale bar: 50 μm.

Immunofluorescence of S-100/NF-200 at the distal end of sciatic nerve injury can detect Schwan Cell (SCs) generation and nerve filament markers, and can evaluate the repair effect of regenerated nerves. Immunofluorescence chemical staining of NF-200/S-100 in each group was shown in Fig. 10. The number of nerve fibers and SCs regeberated in the control group was large the nerve fibers were obviously regenerated along the nerve, and the nerve fibers were more denser, with clear structures and strong fluorescence. Compared with the control group, the fluorescence expression of S-100/NF-200 in model group was weaker, the number of regenerated myelin sheaths and SCs was lower, and the immunofluorescence intensity of S-100 and NF-200 was 0.365 times and 0.581 times lower, respectively, than that of the control group (*P* < 0.05). Myelin regeneration and SCs in the ADSCs group and ADSCs + Res group were better than those in the model group (*P* < 0.05), and myelin regeneration in ADSCs + Res group was higher than that in the ADSCs group. The immune reactivity of S-100 and NF-200 was 0.248 times and 0.125 times higher, respectively, than that of the ADSCs group (*P* < 0.05).

## Discussion

MSCs are important seed cells for repairing tissue damage and promoting tissue regeneration^17,18^. ADSCs are multipotent stem cells that are available in adipose tissueand can be harvested easily through minimally invasive procedures. Studies declared that ADSCs have shown a high cellular yield in culture, and have superior proliferation and differentiation potential than BMSCs. The beneficial effect of ADSCs transplantation on the regeneration of peripheral nerve injuries has been shown in several studies^19,20^. Most of the beneficial effects exerted by MSCs are strongly correlated with the production of neurotrophic substances, such as FGF, NGF, ciliary neurotrophic factor, BDNF and GDNF.^3,21^ ADSCs were injected into rats with sciatic nerve injury. On the 28th day after surgery, we found that the regeneration rate of myelin in the ADSC group was significantly different from that in the model group (p < 0.05).

However, changes in the microenvironment of the injured tissue induced apoptosis of the transplanted MSCs, which weakened the therapeutic efficiency of MSCs^22^. Although the role of ADSCs in peripheral nerve repair has been widely recognized, the low oxygen content and pH value in the microenvironment of the injured tissue make it difficult to maintain the long-term survival of transplanted cells, and most cells undergo apoptosis in the long-term survival process. Some scholars used skin stem cells combined with neural scaffolds to repair peripheral nerve injury, and found that the survival rate of stem cells was less than 10% 2 weeks after transplantation^23^. Toma et al. showed that the survival rate of MSCs transplanted into the heart of immunodeficient mice was less than 0.44% on Day 4^23,24^. To overcome this shortcoming, scholars have adopted a variety of methods to treat or modify MSCs in vitro to improve efficacy, including gene modification and small molecule drug pretreatment^25^. H_2_O_2_ is an important reactive oxygen species. Exogenous H_2_O_2_ can easily cross the cell membrane and enter cells. In the presence of intracellular transition metals the Fenton reaction can form highly active free radicals such as singlet oxygen and hydroxyl radicals, which further cause cell damage^26^, Because of H_2_O_2_’s simple operation and easy control, it is widely used in inducing apoptosis models^27^. To establish a stable and efficient apoptosis model, rat ADSCs were treated with different concentrations of H_2_O_2_ in this study, and we found that with increasing H_2_O_2_ concentration, the cell apoptosis rate gradually increased. The apoptosis rate of ADSCs induced by 300 μmol/L H_2_O_2_ was approximately 50%, which was most suitable for subsequent experiments.

In recent years, the study of small molecule drugs combined with MSCs therapy in tissue injury repair has attracted increasing attention^25^. The main objective is to pretreat MSCs with small molecule drugs in vitro, so that MSCs can obtain stronger proliferation and a higher survival rate in damaged tissues, and ultimately have a better therapeutic effect. The small molecule drug Res is a kind of natural polyphenol plant antitoxin, that widely exists in grape skins, peanuts, mulberrys and other plants and has a variety of biological functions^28^. Res was found to induce the activation of proliferation-related signaling pathways (such as ERK and WNTB-Actenin)^29,30^. Res-preprocessed BMSCs can activate the PI3K/AKT signaling pathway in pancreatic cells and HUVECs through paracrine release of VEGFA; thus, achieving the therapeutic effect of resisting apoptosis of pancreatic cells and promoting regeneration of damaged blood vessels^31^. Peng et al.^32^ found that Res prevented H_2_O_2_-induced retinal stem cell injury by increasing the expression of SirT1 in the retina. In conclusion, Res has the ability to regulate MSCs proliferation. Our results show that after after pretreatment with 10, 20, 40, 80, and 100 μmol/L Res, 300 μmol/LH_2_O_2_ was used to construct an in vitro oxidative stress model. Res protected H_2_O_2_ ADSCs from apoptosis, and 20 μmol/L Res was most effective. Therefore, 20 μmol/L Res was selected to pretreat ADSCs in the subsequent experiments of this study. In this study, we also found that the caspase-3 expression level was significantly upregulated after H_2_O_2_ treatment in ADSCs, and Res pretreatment effectively reversed H_2_O_2_ induction. In recent years, Res has been reported to promote cell proliferation. As reported in these studies, we found that Res at 20 μM not only increased the number of ADSCs but also promoted the proliferation of ADSCs in H_2_O_2_ environment. Therefore, in this study, to avoid apoptosis of ADSCs after transplantation, ADSCs were incubated with Res at 20 μM for 24 h before transplantation and injected into the nerve injury site to treat sciatic nerve injury in rats. We further investigated whether preconditioning ADSCs with Res could optimize the repair effect of ADSCs in rat sciatic nerve injury.

There are many tests evaluating nerve regeneration in laboratory animal models, and they can be evaluated in different ways. We first established sciatic nerve crush injury in rats, a widely used model of peripheral nerve injury. This study evaluated sciatic nerve regeneration in terms of function and histology. Although the ADSCs group and Res + ADSCs group promoted nerve regeneration and functional recovery, the treatment effect of Res + ADSCs group is significantly better than that of the ADSCs group. The study found that Res significantly increased the homing of MSCs to liver and promoted liver regeneration in rats undergoing partial hepatectomy^33^. Res also increases the implantation of MSCs, promotes the repair of hippocampus damage in Alzheimer's disease mice, and alleviates nerve cell apoptosis^34^. The survival rate of ADSCs treated with Res was only increased, and its final fate was the same as that of untreated ADSCs. The recovery of spinal cord motor neuron injury is difficult and it takes a relatively long time. In conclusion, Res-incubated ADSCs play a promoting role in nerve regeneration and functional recovery after sciatic nerve compression injury in rats, which may be related to the increased implantation and survival rate of ADSCs after Res incubation, which needs further study.

After peripheral nerve injury, the change in the microenvironment leads to the decrease in the cell transplantation rate. Res can protect ADSCs from apoptosis. The results of this study showed that Res could better promote nerve regeneration and functional recovery after ADSCs incubation, which may be related to the protective effect of Res on apoptosis of ADSCs cells in vivo. Moreover, statistical analysis showed that the application of ADSCs incubated with Res could significantly improve the quality of nerve repair compared with untreated ADSCs.

## Data Availability

The data supporting this article will be shared on reasonable request to the corresponding author.

## Abbreviations

ADSCs: Adipose derived stem cells
Res: Resveratrol
MSCs: Mesenchymal stem cells
BMSCs: Bone marrow mesenchymal stem cells
SFI: Sciatic nerve function index
SCs: Schwan Cell
